# Metabolomic Response to Acute Hypoxic Exercise and Recovery in Adult Males

**DOI:** 10.3389/fphys.2018.01682

**Published:** 2018-11-26

**Authors:** Gareth Davison, Maria Vinaixa, Rose McGovern, Antoni Beltran, Anna Novials, Xavier Correig, Conor McClean

**Affiliations:** ^1^Sport and Exercise Science Research Institute, Ulster University, Antrim, United Kingdom; ^2^Metabolomics Platform of the Spanish Biomedical Research Center in Diabetes and Associated Metabolic Disorders, IISPV – Rovira i Virgili University, Tarragona, Spain; ^3^Department of Endocrinology, Institut d’Investigacions Biomèdiques August Pi i Sunyer (IDIBAPS), Hospital Clínic de Barcelona, Barcelona, Spain

**Keywords:** metabolomics, exercise, hypoxia, metabolism, purine nucleotide

## Abstract

Metabolomics is a relatively new “omics” approach used to characterize metabolites in a biological system at baseline and following a diversity of stimuli. However, the metabolomic response to exercise in hypoxia currently remains unknown. To examine this, 24 male participants completed 1 h of exercise at a workload corresponding to 75% of pre-determined 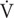
O_2max_ in hypoxia (F_i_o_2_ = 0.16%), and repeated in normoxia (F_i_o_2_ = 0.21%), while pre- and post-exercise and 3 h post-exercise metabolites were analyzed using a LC ESI-qTOF-MS untargeted metabolomics approach in serum samples. Exercise in hypoxia and in normoxia independently increased metabolism as shown by a change in a combination of twenty-two metabolites associated with lipid metabolism (*p* < 0.05, pre vs. post-exercise), though hypoxia *per se* did not induce a greater metabolic change when compared with normoxia (*p* > 0.05). Recovery from exercise in hypoxia independently decreased seventeen metabolites associated with lipid metabolism (*p* < 0.05, post vs. 3 h post-exercise), compared with twenty-two metabolites in normoxia (*p* < 0.05, post vs. 3 h post-exercise). Twenty-six metabolites were identified as responders to exercise and recovery (pooled hypoxia and normoxia pre vs. recovery, *p* < 0.05), including metabolites associated with purine metabolism (adenine, adenosine and hypoxanthine), the amino acid phenylalanine, and several acylcarnitine molecules. Our novel data provides preliminary evidence of subtle metabolic differences to exercise and recovery in hypoxia and normoxia. Specifically, exercise in hypoxia activates metabolic pathways aligned to purine and lipid metabolism, but this effect is not selectively different from exercise in normoxia. We also show that exercise *per se* can activate pathways associated with lipid, protein and purine nucleotide metabolism.

## Introduction

Metabolomics is a comprehensive and quantitative analysis of all metabolites in a biological system, that has enhanced our understanding of the physiological response to internal and environmental stressors, by permitting the acquisition of a “snapshot” of a whole organism’s metabolic status at a given moment. Whilst the benefits of exercise for health are widely recognized, the underpinning mechanisms are still not entirely clear. A limited number of studies have described the challenge of exercise to the human metabolome in an effort to provide further mechanistic insights (Enea et al., 2010; Lewis et al., 2010; Pechlivanis et al., 2010; Brugnara et al., 2012). These studies have largely confirmed and extended previous theories on exercise-induced metabolic and molecular adjustments (Enea et al., 2010; Pechlivanis et al., 2010), but have also highlighted, among others, the role of exercise intensity on mediators of substrate utilization and the potential biological benefits to muscle tissue of compounds such as acylcarnitines (Lehmann et al., 2010). Moreover, an elevation in products of purine metabolism, such as adenosine, hypoxanthine, inosine, uric acid and xanthine, in the post-exercise metabolome, have also been identified. Such findings are clearly of interest given their putative roles in cell signaling following exercise-induced changes in reactive oxygen species (ROS) and metabolism (Hellsten et al., 1999; Lewis et al., 2010; Pechlivanis et al., 2010).

Hypoxia is a common stressor utilized to examine physiological, biochemical and molecular responses stemming from changes in both systemic and cellular oxygen availability (Murray, 2009). Hypoxia depletes ATP stores, increases cellular metabolic stress at rest and during acute exercise (with a subsequent reliance on anaerobic metabolism), and decreases work capacity compared with normoxic states (Heinonen et al., 2012; Morales-Alamo et al., 2012). It is also known that exercise in hypoxia can elicit oxidative stress following acute exposure (Davison et al., 2006), whereas more prolonged exposure can attenuate this response (Debevec et al., 2014). Furthermore, the metabolic adaptations that occur with exercise training in hypoxia seem to differ from those observed (in exercise) in normoxia (Desplanches et al., 1996; Bassett and Howley, 2000), and such discrepancies may relate to hypoxic variations in exercise-mediated gene transcription (De Palma et al., 2007; Hoppeler et al., 2008). Analysis of rat muscle exposed to chronic hypoxia has shown a decrease in ATP generating pathways, TCA cycle intermediates and an increase in glycolytic enzymes (De Palma et al., 2007). Furthermore, metabolomic analysis of *drosophilae* (fruit flies) has identified that tolerance to hypoxia is dependent upon both the degree of homeostatic pH control and ability to maintain ATP production (Feala et al., 2007). As such, ATP/substrate ratio, in addition to markers of purine catabolism, may provide an insight to hypoxic tolerance (Hochachka et al., 2002; Feala et al., 2007).

Recent work has documented metabolomic changes following exposure to hypoxia with concurrent restrictions to physical activity in healthy volunteers (Šket et al., 2018). To date however, no study has examined the role of exercise in hypoxia using metabolomics profiling in a human model. An exploration of the metabolomic responses to exercise in moderate hypoxia is necessary, particularly when considering the ability of metabolomics to provide a global analysis of metabolites and metabolic patterns that could provide salient insights to genomic and proteomic changes (Serkova et al., 2008). The aim of the present study therefore, is to quantify the degree of change to metabolism following a single bout of exercise in human participants exposed to moderate hypoxia using LC ESI-qTOF-MS untargeted metabolomics.

## Materials and Methods

### Human Participants and Experimental Design

Following ethical approval from a local Research Ethics Committee, twenty-four (*n* = 24) apparently healthy male participants were recruited. All participants were considered trained, and completed moderate to high intensity aerobic exercise of 60 min or more 4–6 times weekly. A standard institutional lifestyle and health-screening questionnaire identify and excluded any participant who had a history of metabolic or cardio-pulmonary disorder, epilepsy, sleep apnoea, or a history of smoking or alcohol/drug abuse. Individuals were also excluded if they experienced chronic or intermittent altitude exposure for more than 3 weeks in the 6 weeks prior to the beginning of the study. Before commencement, participants provided written informed consent. Participants continued with their normal diet and exercise habits for the duration of the study, with the exception that participants were requested to use the day preceding testing as a rest day, and to arrive for steady-state exercise following an 8 h over-night fast. All participants attended a familiarization session 1 week prior to the commencement of the incremental test protocol.

### Anthropometric Measures

Body mass in kilograms (kg) was recorded to the nearest 0.1 kg using standard scales (Seca delta, Germany). Stature was recorded to the nearest 0.1 cm using a freestanding stadiometer (Holtain Limited, Great Britain). See Table 1 for further details of participant anthropometric data.

**Table 1:** Participant characteristics.

Age (years)	28 ± 5
Stature (cm)	177 ± 6
Mass (kg)	74 ± 8
Resting heart rate (bpm)	59 ± 13

*All data are expressed as a mean ± SD.*

### Experimental Design and Incremental Test Protocol

**FIGURE 1:**
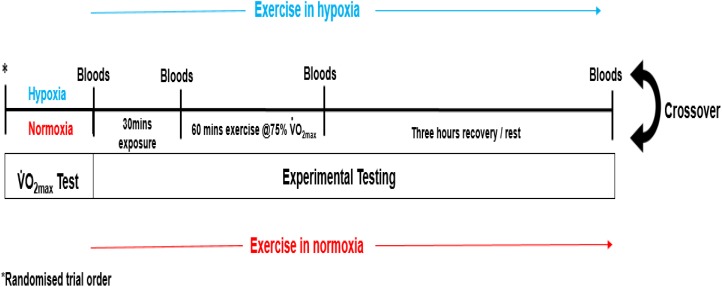
Experimental design.

Each participant completed a double-blinded randomized controlled crossover trial (see Figure 1). Prior to experimental testing, participants completed two incremental treadmill tests to volitional exhaustion (10–13 min typical duration), one in moderate hypoxia (inspired fraction of oxygen {F_i_o_2_} = 0.16%) and the other in normoxia (F_i_o_2_ = 0.21%). The environmental and subsequent exercise condition for participants, was achieved through the use of a hypoxic chamber (Design Environmental Ltd., Wales), in combination with Contour programming and a logging software package (Contour software, Design Environmental Ltd., Wales) to set and monitor the desired oxygen availability through adjustments in nitrogen in-flow. Each test was separated by 7 days. The treadmill test consisted of an initial set exercise speed of 8–10 km/h with a 0% gradient. Work rate was increased by 1 km/h every 90 s until volitional exhaustion. This protocol was chosen as it had previously been used to elicit 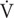
O_2max_ in hypoxia (Dufour et al., 2006). Expired gas was continuously monitored using an online gas analysis system (Quark, CPET, Cosmed, Italy) while rate of perceived exertion (RPE; Borg Scale), lactate (Lactate Pro, Arkray, Japan) and heart rate (HR; Polar telemetry device, RS800, Sweden) were recorded at the end of each stage. The data was used to calculate work rate corresponding to 75% 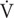
O_2max_ for each participant.

### Experimental Protocol

On arrival at the laboratory, participants rested in the supine position while baseline samples of capillary oxygen saturation (SpO_2_) using a finger pulse oximeter (Merlin M-scope, Medscope, United Kingdom) and HR were obtained. Each participant then commenced a further 30 min rest in hypoxia (F_i_o_2_ = 0.16%) or normoxia (F_i_o_2_ = 0.16%) after which secondary measures of SpO_2_ and primary measures of venous blood were obtained. Participants subsequently completed a double-blind, randomized, cross-over trial, where each participant completed 1 h of submaximal exercise on a motorized treadmill, at a workload corresponding to 75% of pre-determined 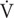
O_2max_ in hypoxia (F_i_o_2_ = 0.16%), and subsequently 75% of pre-determined 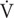
O_2max_ in normoxia (F_i_o_2_ = 0.21%), respectively. Throughout steady-state exercise, HR and expired gas were measured using an online gas analysis system. At 15 min intervals, RPE and SpO_2_ was recorded. On immediate cessation of exercise, participants remained in the environmental chamber, and in the supine position provided a post-exercise venous blood sample. Venous blood samples were also obtained 3 h post-exercise. All exercise testing was performed between the hours of 08:00 and 10:00.

### Hematology

Blood samples were obtained from a prominent antecubital forearm vein using the Vacutainer method, and dispensed into serum separating (SST) tubes, and allowed to clot at room temperature. All samples were centrifuged at 3500 rpm for 10 min prior to storage at −70°C and subsequent analysis. Arterialized finger capillary blood was obtained using a lancing device (Accu-check, West Sussex, England, United Kingdom) for the determination of haematocrit (Hct) and haemoglobin (Hb). Hct was measured following centrifugation using a micro haematocrit reader (Hawksley, London, United Kingdom), while hemoglobin was measured using a hemocue analyzer (Hemocue, Derbyshire, United Kingdom) inter- and intra-assay CV < 3% for both Hct and Hb. The known concentrations of Hb and Hct were used to calculate post-exercise changes in plasma volume using the method of Dill and Costill (1974).

### Untargeted LC-MS Metabolomic Profiling

An untargeted metabolomic approach was used to assess the metabolomic profile of serum samples (Patti et al., 2012). This method incorporated liquid chromatography coupled to electrospray ionization quadruple time-of-flight mass spectrometry (LC ESI-qTOF-MS). Metabolites were extracted as follows: 50 μL of serum was mixed with 400 μL of MeOH/H_2_O (8:1 v/v) containing 1% formic acid. Samples were vortexed vigorously for 30 s and stored at 20°C for 2 h to enable protein precipitation. Subsequently, samples were centrifuged at 15,000 rpm for 15 min at 4°C and the supernatant was transferred to a LC-MS vial. Samples were injected into a UHPLC system (1290 Agilent) coupled to a quadruple time of flight (QTOF) mass spectrometer (6550 Agilent Technologies), operated in either positive or negative electrospray ionization modes (ESI+/ESI-). When the instrument was operated in positive ionization mode, metabolites were separated using an Acquity UPLC (HSS T3) C18 reverse phase (RP) column (2.1 × 150 mm, 1.8 μm) and the solvent system was A1 = 0.1% formic acid in water and B1 = 0.1% formic acid in acetonitrile. When the instrument was operated in negative ionization mode, metabolites were separated using an Acquity UPLC (BEH) C18 RP column (2.1 × 150 mm, 1.7 μm) and the solvent system was A2 = 1 mM ammonium fluoride in water and B2 = acetonitrile. The linear gradient elution started at 100% A (time 0–1.5 min) and finished at 100% B (12–15 min). The injection volume was 1 μL. ESI conditions were gas temperature, 290°C; drying gas, 13 L × min^−1^; nebulizer, 35 psig; fragmentor, 120 V; and skimmer, 65 V. The instrument was set to acquire over the *m*/*z* range 60–1000 with an acquisition rate of 3 spectra/s. Quality control samples (QCs) consisting of pooled serum samples were used to assess analytical variability. QCs were injected periodically throughout the work list after 5-study samples. Samples entering the study were entirely randomized to reduce systematic error associated with instrumental drift.

### Statistical Analysis

Prospective power calculations identified a sample population of 24 participants based on comparison of standardized means. The equation N = $\frac{4\sigma^{2}{\left(\mathrm{zc}+\mathrm{zp}\right)}^{2}}{D^{2}}$ was used where D = effect size, σ = SD (root mean squared), z_c_ a constant for alpha level 0.05 and z_p_ a constant for a desired power of 95% (Rosner, 2010). The physiological variables measured during the maximal oxygen uptake tests were analyzed using an unpaired samples *t*-test.

LC-MS (RP-C18 ESI+ and ESI- mode) data were processed using the XCMS software (version 1.38.0) (Smith et al., 2006) to detect and align features. A feature is defined as a molecular entity with a unique m/z and a specific retention time. XCMS analysis of these data provided a matrix containing the retention time, m/z value, and integrated peak area of each feature for every sample. QC samples were used to filter analytical variation. Features were log-transformed and analyzed by two-way ANOVA with exercise and hypoxia as main factors. Significant interactions were followed by Tukey’s HSD *post hoc* comparisons to assess the differences between groups. Pooled data represents main effect for time (pooled hypoxic and normoxic data at pre vs. post vs. 3 h recovery) analysis. False discovery rate was controlled using the Benjamini and Hochberg (1995) procedure. Differentially regulated features that passed statistical criteria (*p* < 0.05) were characterized by LC-qTOF MS/MS (Brugnara et al., 2012). MS/MS was performed in targeted mode, and the instrument was set to acquire over the *m/z* RANGE 50–1000, with a default iso width (the width half-maximum of the quadruple mass band pass used during MS/MS precursor isolation) of 4 *m/z*. The collision energy was fixed at 20 V. For identification purposes MS/MS spectra were compared against Metlin (Smith et al., 2006) or HMDB (Wishart et al., 2013) databases.

## Results

### Physiological Response to Maximal Exercise

There was full adherence to the study (100%). Maximal aerobic capacity was greater in normoxia in comparison to the test performed in hypoxia (*p* < 0.05). Furthermore, post-exercise oxygen saturation was lower (-3.8%) in the hypoxia condition (*p* < 0.05 vs. normoxia, see Table 2).

**Table 2:** Physiological response to exercise in hypoxia (*n* = 24) and normoxia (*n* = 24).

	Hypoxia	Normoxia
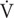 O_2max_ (ml.kg^−1^.min^−1^)	45.8 + 2	60 + 9^∗^
Lactate max (mmol.L)	10.5 + 2	9.7 + 3
Heart rate max (bpm)	185 + 8	189 + 10
RPE max (arbitrary units)	19 + 0	20 + 0
Speed max (km/h)	18.2 + 4	18.5 + 1
SpO_2_ (%)	93	97^∗^

*All data are expressed as a mean ± SD. ^∗^Different from hypoxia, p < 0.05.*

### Metabolomic Profiling as a Function of Exercise and Recovery in Hypoxia vs. Normoxia

There were no selective differences in metabolites between the trials following exercise (normoxia vs. hypoxia *p* > 0.05). Furthermore, there was no difference in metabolism following hypoxic/normoxic exposure 3 h into recovery (normoxia vs. hypoxia *p* > 0.05). However, a within trial change was detected whereby exercise performed in hypoxia alone independently increased metabolism as shown by a change in seventeen metabolites associated with acylcarnitine (*p* < 0.05 pre- vs. post-exercise) and five metabolites associated with fat oxidation (*p* < 0.05 pre- vs. post-exercise) as shown in Tables 3, 4. Butyrylcarnitine did not change post-exercise in hypoxia, but increased in the normoxia only trial (*p* < 0.05 vs. post-exercise). Recovery from exercise in hypoxia independently decreased a total of seventeen metabolites aligned with acylcarnitine (*n* = 13) and the fatty acid response (*n* = 4) (*p* < 0.05 vs. 3 h post-exercise), while recovery from exercise in normoxia independently decreased 22 metabolites (*p* < 0.05 vs. 3 h post-exercise) as shown in Tables 3, 4.

**Table 3:** Acylcarnitine response to exercise and recovery in hypoxia and normoxia.

Secreted metabolites	Exercise (pre- to post-exercise)		Recovery (post- to 3 h post-exercise)	
	Hypoxia	Normoxia	Hypoxia	Normoxia
Propionylcarnitine	1.69^∗^	1.88^∗†^	−2.10^‡^	−2.49^‡†^
Butyrylcarnitine	1.60	1.95^∗†^	−2.13^‡^	−2.46^‡†^
2-Methylbutyroylcarnitine/Pivaloylcarnitine	1.75^∗^	2.00^∗†^	−1.98^‡^	−2.50^‡†^
Hexanoylcarnitine	3.82^∗^	4.54^∗†^	−2.70^‡^	−2.88^‡†^
Octanoylcarnitine	2.95^∗^	3.71^∗†^	−2.28	−2.62^‡†^
Decanoylcarnitine	3.08^∗^	3.65^∗†^	−2.20	−2.56^‡†^
Undecenoylcarnitine	2.13^∗^	2.40^∗†^	−1.55	−1.90^‡†^
4,8 dimethylnonanoyl carnitine	2.81^∗^	3.14^∗†^	−2.79^‡^	−3.66^‡†^
Dodecenoylcarnitine	2.42^∗^	2.43^∗†^	−1.62^‡^	−1.79^‡†^
Tridecenolycarnitine	1.91^∗^	2.21^∗†^	−1.70^‡^	−2.04^‡†^
Tetradecadienoylcarnitine	3.77^∗^	4.17^∗†^	−2.48^‡^	−2.67^‡†^
cis-5-Tetradecenoylcarnitine	3.86^∗^	4.31^∗†^	−2.37^‡^	−2.23^‡†^
Hexadecatetraenoylcarnitine	2.58^∗^	3.09^∗†^	−2.39^‡^	−2.37^‡†^
Hexadecadienoylcarnitine	3.83^∗^	4.81^∗†^	−3.24^‡^	−3.01^‡†^
Hexadec-2-enoyl carnitine	3.44^∗^	3.89^∗†^	−2.29^‡^	−2.12^‡†^
Palmitoylcarnitine	1.61^∗^	1.53^∗†^	−1.14	−1.18
Octadecatrienoylcarnitine	2.03^∗^	2.15^∗†^	−1.84^‡^	−1.81^‡†^
Elaidic carnitine/Vaccenyl carnitine	1.96^∗^	1.94^∗†^	−1.47	−1.41^†^

*All data expressed as a fold change. ^∗^Difference between pre vs. post-exercise, p < 0.05; ^‡^difference between post-exercise vs. 3 h post-exercise, p < 0.05; ^†^indicates main effect for time (pooled hypoxia and normoxia values pre vs. post-exercise vs. 3 h post-exercise, p < 0.05).*

**Table 4:** Hippuric and fatty acid response to exercise and recovery in hypoxia and normoxia.

Secreted metabolites	Exercise (pre- to post-exercise)		Recovery (post- to 3 hr post-exercise)	
	Hypoxia	Normoxia	Hypoxia	Normoxia
Hippuric acid	1.37	2.10	−2.65	−2.98^‡†^
Myristic acid	1.83^∗^	1.99^∗†^	−1.45^‡^	−1.53^‡†^
Palmitoleic acid	4.28^∗^	5.36^∗†^	−1.95	−2.06^‡†^
α-Linolenic acid	3.50^∗^	4.67^∗†^	−2.01^‡^	−2.12^‡†^
Linoleic acid	2.70^∗^	3.17^∗†^	−1.62^‡^	−1.79^‡†^
Arachidonic acid	2.15^∗^	2.37^∗†^	−1.68^‡^	−1.63^‡†^

*All data expressed as a fold change. ^∗^Difference between pre vs. post-exercise, p < 0.05; ^‡^difference between post-exercise vs. 3 h post-exercise, p < 0.05; ^†^indicates main effect for time (pooled hypoxia and normoxia values pre vs. post-exercise vs. 3 h post-exercise, p < 0.05).*

### Metabolomic Profiling as a Function of Exercise and Recovery *per se*

Twenty-seven metabolites were identified as either increasing or decreasing as a function of exercise and recovery (pooled data pre vs. post vs. recovery, *p* < 0.05). These include metabolites associated with purine metabolism (adenine, adenosine and hypoxanthine), the amino acid, phenylalanine, several acylcarnitines and some unknown metabolites.

### Purine Metabolism

**FIGURE 2:**
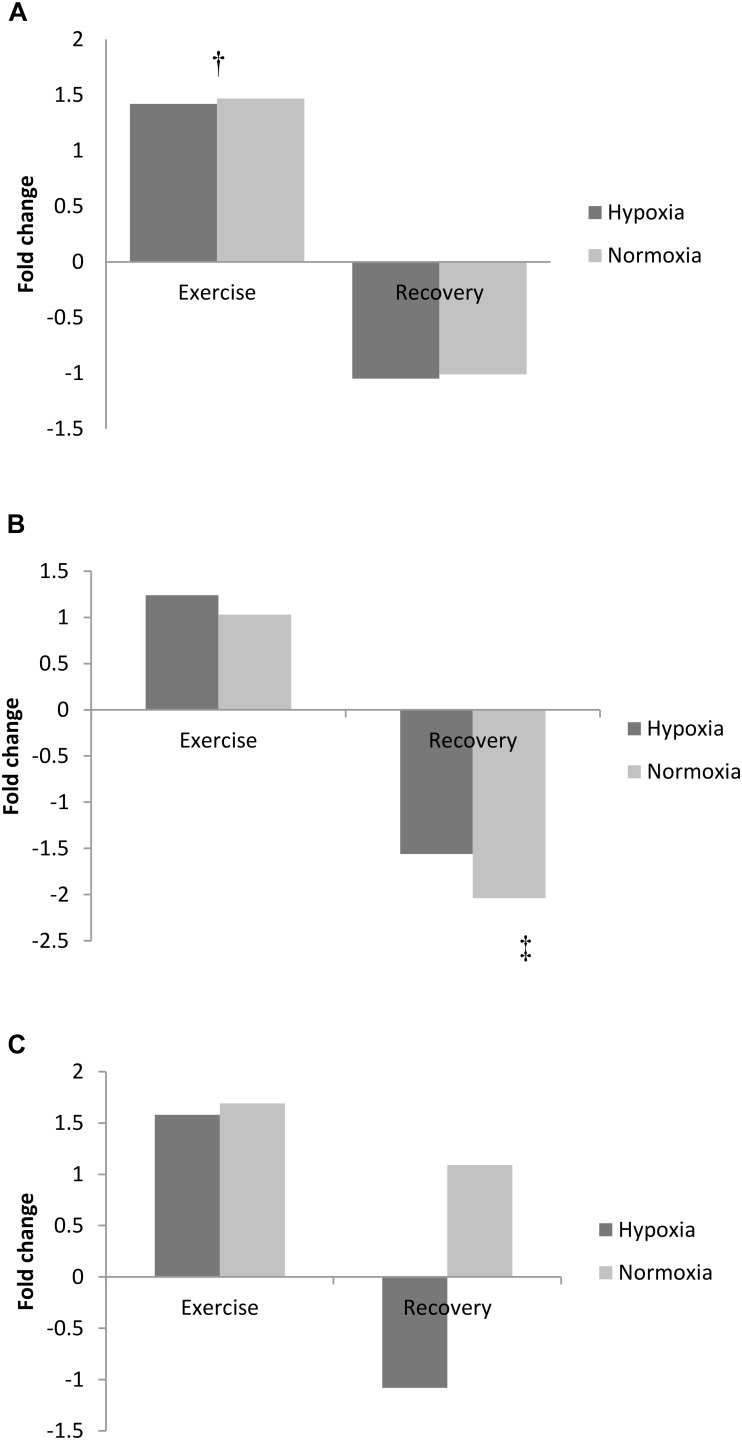
Exercise and recovery for **(A)** adenine, **(B)** hypoxanthine, and **(C)** adenosine in hypoxia and normoxia. All values expressed as a fold change. ^‡^Difference between post-exercise vs. 3 h post-exercise, *p* < 0.05; ^†^indicates main effect for time (pooled hypoxia and normoxia values for pre vs. post-exercise) *p* < 0.05.

Exercise increased adenine concentration (pooled data pre- vs. post-exercise, *p* < 0.01) and was still elevated 3 h post-exercise (pooled data pre- vs. 3 h recovery, *p* < 0.01; Figure 2A). Adenosine increased from pre-exercise to 3 h recovery post-exercise (pooled data, *p* < 0.01; Figure 2B). In contrast to adenine and adenosine, hypoxanthine concentration was unchanged by exercise but markedly decreased following 3 h recovery (pooled data exercise vs. 3 h recovery, *p* < 0.05, Figure 2C).

### Phenylalanine Metabolism

**FIGURE 3:**
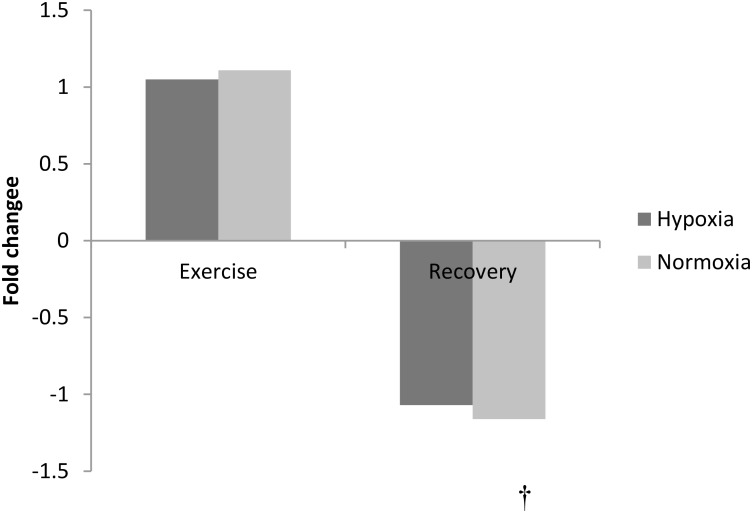
Exercise and recovery for phenylalanine in hypoxia and normoxia. All values expressed as a fold change. ^†^indicates main effect for time (pooled hypoxia and normoxia values for post-exercise vs. 3 h post-exercise) *p* < 0.05.

The amino acid phenylalanine decreased at 3 h recovery following exercise (pooled data, *p* < 0.01, Figure 3). No other amino acid changed with exercise or in recovery (*p* > 0.05).

### Acylcarnitine and Fatty Acid Metabolism

Exercise significantly increased eighteen acylcarnitine metabolites (pooled pre vs. post-exercise, *p* < 0.01, Table 3) by as much as 4.3-fold. The majority of acylcarnitines decreased following exercise but remained above pre-exercise values at 3 h recovery (pooled post-exercise vs. 3 h post-exercise, *p* < 0.05, Table 3). Six metabolites were identified as fatty acid intermediaries, and five increased immediately post-exercise (pooled pre- vs. post-exercise, *p* < 0.01, Table 4), while there was no change for hippuric acid (*p* > 0.05). All fatty acids decreased during recovery (pooled post-exercise vs. 3 h post-exercise, *p* < 0.05, Table 3).

### Unknown Metabolites as a Function of Hypoxia and Exercise

There were changes in several unnamed metabolites that are likely to be phospholipid in origin, and this supposition is based on the carbon structure of the molecule. Exercise in hypoxia did not selectively change the metabolomic response to any unknown metabolite when compared with normoxia (*p* > 0.05). There was also no difference in metabolism following hypoxic exposure 3 h into recovery (*p* > 0.05 vs. normoxia). However, exercise performed in hypoxia and normoxia independently increased PC (16:0/0:0) metabolism (*p* < 0.05 vs. post-exercise) as shown in Table 5. Furthermore, exercise *per se* increased the concentration of three unknown metabolites (pooled pre- vs. post-exercise, *p* < 0.05, Table 5).

**Table 5:** Unknown metabolites following exercise and recovery in hypoxia and normoxia.

Secreted metabolites	Exercise (pre- to post-exercise)		Recovery (post- to 3 h post-exercise)	
	Hypoxia	Normoxia	Hypoxia	Normoxia
PE (18:1/0:0)	−1.26	−1.31^†^	−1.15	−1.18
PC (16:0/0:0)	−1.24^∗^	−1.27^∗†^	1.09	1.05
PC (18:3/0:0)	−1.15	−1.18	−1.23	−1.28
PC (18:2/0:0)	−1.24	−1.27^†^	−1.13	−1.19
PC (16:0/2:0)-[M+H-H_2_O]	−1.09	−1.08	−1.09	−1.13
PC (O-16:1/2:0)/PC(18:1/0:0)	−1.15	−1.18	−1.05	−1.08
LPE (22:6/0:0)	−1.03	−1.07	1.32	1.34

*All data is expressed as a fold change. ^∗^Difference between pre vs. post-exercise, p < 0.05; ^†^indicates main effect for time (pooled hypoxia and normoxia values for pre vs. post-exercise, p < 0.05).*

## Discussion

This study used an untargeted metabolomics approach to provide insight into the biochemical effects of exercise in hypoxia. To the best of our knowledge, this is the first preliminary report on the metabolomic response to exercise in hypoxia. Our findings demonstrate a similar metabolic pattern following exercise in hypoxia when compared with normoxia. However, exercise in hypoxia does not selectively change metabolism at the circulating level, which may relate to the moderate hypoxia paradigm used. We identify several novel metabolites with unknown functionality, but given that metabolomic profiling is a relatively new “-*omics*” approach within exercise physiology research, we hope that further work extends our preliminary findings.

Substantial changes in several metabolites were observed in response to exercise *per se*, suggesting an active utilization of fuel substrates in several metabolic pathways including acylcarnitines and fatty acids; markers that are regularly used as indicators of metabolic disorders (Barderas et al., 2011). Markers of purine metabolism indicative of nucleotide degradation were also elevated following exercise. The pattern of metabolic intermediates observed was similar to that normally associated with maximal, energy depleting exercise (Balsom et al., 1992), whilst other investigators using targeted (Lewis et al., 2010) and untargeted (Lehmann et al., 2010) metabolomic analyses show similar changes in intermediates of purine catabolism (Lewis et al., 2010), fatty acids (Lewis et al., 2010; Lehmann et al., 2010), and acylcarnitines (Lehmann et al., 2010) following exercise in humans.

### Fatty Acid and Intermediates of Fatty Acid Transport (Acylcarnitines)

Fatty acids are a high-energy source for mitochondrial ATP generation (Jeppesen and Kiens, 2012). Aerobic exercise can cause an increase in systemic fatty acid mobilization (Horowitz, 2003), enhance intracellular and mitochondrial transport (Bonen et al., 2000; Jeppesen and Kiens, 2012) and increase fat oxidation (Bradley et al., 2012). In accordance with published literature, metabolomic analysis revealed up to a fourfold increase in fatty acid concentration as a function of exercise. Furthermore, exercise increased several acylcarnitines, which are low molecular weight compounds essential for the transport of long chain fatty acids across the mitochondrial membrane (Houten and Wanders, 2010; Furuichi et al., 2014). Acylcarnitines are the product of short (<5 carbons), moderate (6–12 carbons) and long chain fatty acids (13–21 carbons) produced in fatty acid degradation (Ventura et al., 1998). Due to their small length and solubility, acylcarnitines can move across the mitochondrial membrane where they contribute to the formation of acyl-CoA for the TCA cycle via the carnitine shuttle (Houten and Wanders, 2010; Furuichi et al., 2014). Acylcarnitines also work in reverse, in response to elevated intracellular fatty acids to prevent the accumulation of acyl-coenzyme-A and concomitant retardation of mitochondrial metabolism (Furuichi et al., 2014). Either way, elevated acylcarnitines reflect an increase in the availability of fatty acid and potential for mitochondrial fatty acid oxidation (Lehmann et al., 2010; Jeppesen and Kiens, 2012).

During sustained exercise, utilization of fatty acids for energy generation is associated with improved and prolonged exercise capacity (Kiens et al., 1993). It is known that supplementation with propionylcarnitine, carnitine and acylcarnitine preserve muscle glycogen, and can off-set lactate accumulation, suggesting that acylcarnitine may mediate the rate of fatty acid oxidation during exercise (Morand et al., 2014). Therefore, the increase in acylcarnitine in addition to the other lipid metabolites observed in the current study, may be illustrative of an exercise-induced increase in fatty acid transport to enhance mitochondrial metabolic capacity in moderate aerobic exercise. Although, values decreased during the recovery period, these were still elevated above pre-exercise values, and as such, transient changes may be important triggers for improvements in fat metabolism consistent with endurance training (Jeppesen and Kiens, 2012). Nevertheless, there was no difference in acylcarnitines observed between hypoxic and normoxic states, and this was despite the decrease in fat utilization normally demonstrated in hypoxia (Ozcelik et al., 2003). Based on our preliminary data, we postulate that acylcarnitine-mediated transport of fatty acids into the mitochondria is unaffected by oxygen availability. Further work is required to ascertain the proposed role of acylcarnitine and its relationship with intracellular fatty acid transport.

### Purine Metabolism

Although there is a considerable body of evidence depicting adenosine as the primary cause of tissue vasodilation in both ischemia and hypoxia (MacLean et al., 1998; Walsh and Marshall, 2006), we did not observe a selective change in adenosine following exercise in hypoxia, although this may be due to the relatively short hypoxic exposure; recent evidence by Strewe et al. (2018) reports a change in adenosine concentration following a more prolonged hypoxic exposure. Moreover, we did not observe changes in any associated marker of purine metabolism with exercise in hypoxia. However, exercise *per se* increased the serum content of adenine and its nucleoside product adenosine, suggestive of nucleotide degradation (Hargreaves, 2000), and this increase was still evident 3 h following exercise in the recovery period. Previous studies have reported an increase in adenosine and adenine (normally as adenine nucleotides) following high intensity exercise (Balsom et al., 1992; Zieliński and Kusy, 2012).

As submaximal exercise was utilized within the current study, it is likely that this intensity was insufficient for ATP degradation to exceed the rate of synthesis within skeletal muscle (Sahlin et al., 1998; Hargreaves, 2000), and this is supported by the lack of upstream change in hypoxanthine. Furthermore, while adenosine and adenine increased, this was not accompanied by AMP deamination and inosine production, which is typical of the anaerobic nucleotide catabolic pathway (Hellsten et al., 1999). Moreover, a decrease in the efflux of purines during acute exercise, limiting muscle nucleotide loss, has been reported in trained individuals (Hellsten-Westing et al., 1993). Mitochondria from active skeletal muscle show an increase in adenine concentration in response to heightened intracellular Ca^2+^ which, during exercise, is associated with an increase in glucagon (Amigo et al., 2013). This suggests that skeletal muscle may be a sink rather than a source of adenosine during contraction (Lynge et al., 2001). Therefore, it is likely that the observed increase in adenine and adenosine molecules following exercise did not originate from the degradation of muscle nucleotides. Rather, it is possible that the increase in free plasma adenosine and adenine arose from extracellular sources (Chu et al., 2013), vascular cells (Marshall, 2000) or possible degradation of liver adenosine nucleotides (Bontemps et al., 1983). Conversely, Heinonen et al. (2011) suggests that adenosine is likely produced in a cell signaling capacity to enhance metabolism associated with respiring muscle cells at low to moderate exercise intensities. It is tentatively suggested that the effects of adenosine and adenine may be hormetic in nature; exercise-induced changes within a physiological range for possible vasodilatory, ATP synthesis and glucose uptake effects (Zieliński and Kusy, 2012).

### Phenylalanine

Phenylalanine is an essential amino acid, and one of the large neutral amino acids required for the synthesis of protein (Matthews, 2007). It is involved in the anabolism of catecholamines, epinephrine, norepinephrine, and dopamine, which in turn are involved in the moderation of energy producing pathways (Fernstrom and Fernstrom, 2007). Because phenylalanine is neither oxidized nor synthesized endogenously, a change in concentration is indicative of a change in protein synthesis or degradation (Børsheim et al., 2002). Our findings demonstrate that phenylalanine decreases within the recovery phase of 1 h of moderate intensity exercise, and this may be indicative of an increased rate of protein degradation as a function of exercise (Matthews, 2007). Several other essential amino acids are involved in protein synthesis and degradation, however, these, unlike phenylalanine, are susceptible to oxidation (e.g., leucine) (Børsheim et al., 2002). Given that there were only few significant main effects observed for metabolites associated with fuel oxidation, it is likely that fuel oxidation was variable between participants; an affect that may have extended to the amino acid metabolic pathways (Lewis et al., 2010).

### Unknown Metabolites

As indicated previously, several metabolites detected are currently unknown, and three in particular changed following exercise, while there was no selective difference in hypoxia for any aspect of metabolism. It is likely that most of these detected metabolites are phospholipid in origin, but this needs to be categorically confirmed. To offer any detailed biochemistry in explanation of the changes observed at this juncture would be purely speculative; clearly further parallel work is warranted.

## Conclusion

To our knowledge, this is the first study of its kind to ascertain the effect of exercise in moderate hypoxia on the metabolomic response in endurance-trained males. However, in contrast to our original hypothesis, our work demonstrates that exercise in moderate hypoxia *per se* does not differentially change metabolism. There were, however, substantial changes in several metabolites in response to exercise (combined normoxic and hypoxic trials). Further work is required to characterize and ascertain the origin of the unknown metabolites that were shown to augment following exercise, and indeed it would be interesting and salient to examine the metabolic response to exercise using a more severe inspired fraction of oxygen (e.g., *circa* 12%). It may also be of interest to characterize the exercise response across a sample that is more representative of the general population, and this would serve to determine whether any observed changes are linked to specific health and fitness parameters.

## Ethics Statement

Following ethical approval twenty-four healthy male participants were recruited. This study was carried out in accordance with the recommendations of the guidelines as outlined within Ulster University Research Ethics Committee. The protocol was approved by the Ulster University Research Ethics Committee. All subjects gave written informed consent in accordance with the Declaration of Helsinki.

## Author Contributions

GD, RM, CM, and AN contributed to conception and design of the study. MV, XC, and AB performed the biochemical and statistical analysis. GD, RM, and CM wrote the first draft of the manuscript. All authors contributed to manuscript revision, read and approved the submitted version.

## Conflict of Interest Statement

The authors declare that the research was conducted in the absence of any commercial or financial relationships that could be construed as a potential conflict of interest.
